# Developing institution-specific admission competency criteria for prospective health sciences students

**DOI:** 10.1186/s12909-024-06495-8

**Published:** 2024-12-18

**Authors:** Eunhee Kang, Ryan Jin Young Kim, Young-Seok Park, Shin-Young Park, Jihyun Lee

**Affiliations:** 1 Center for Future Dentistry, School of Dentistry, Seoul National University, Seoul, Republic of Korea; 2 Department of Dental Education, School of Dentistry & Dental Research Institute, Seoul National University, 103 Daehak-ro, Jongno-gu, Seoul, 03080 Republic of Korea; 3 Department of Oral Anatomy, School of Dentistry & Dental Research Institute, Seoul National University, Seoul, Republic of Korea

**Keywords:** Admission competency, Health sciences education, Healthcare professional, Evidence-based mission-driven selection processes

## Abstract

**Background:**

Given the critical role of health professionals in societal health, the development of robust and effective selection methods is of fundamental concern for educational institutions within the field of health sciences education. Conventionally, admission competencies have been determined by institutional authorities. Developing institution-specific competency criteria enables an admission process that is mission- and value-aware, evidence-based, and strategically adaptable. However, few schools have established their admission competency criteria, although the majority possess their own models of graduation competencies. This study reports the process of developing and validating an institution-specific admission competency model that addresses the need for evidence-based and mission-aligned selection processes that are distinct from standardized models.

**Methods:**

This study was conducted in two phases, using both qualitative and quantitative analyses. Phase I involved constructing an admission competency model through a qualitative approach facilitated by workshops with 17 faculty members and 92 first-year pre-doctoral students of a dental school. Through constant comparative analysis, this phase focused on the extraction and refinement of competencies for entering dental students. In Phase II, a questionnaire developed from the workshops asked respondents to rate the importance of 47 attributes across 10 constructs on a 5-point Likert scale. A total of 301 individuals participated in the survey. Exploratory Factor Analysis (EFA) identified the factor structure, and Confirmatory Factor Analysis (CFA) examined construct validity and assessed the model fit with the data.

**Results:**

The EFA of the 47 attributes identified 10 factors, and the CFA results indicated a good-to-acceptable level of fit for the ten-factor model. Aligned with the American Association of Medical Colleges Premed competencies, this study identified unique attributes specific to the institution, such as confidence, leadership, and entrepreneurship. These findings highlight the importance of developing tailored competencies reflecting the unique needs of institutions and their fields.

**Conclusions:**

This study demonstrates the feasibility and value of creating institution-specific admission competency models, offering a methodology that aligns with evidence-based mission-driven selection processes. The distinct competencies identified emphasize the need for educational institutions to consider unique institutional and field-specific requirements and move beyond standardized models to enhance the selection of medical students.

## Introduction

Identifying the qualities of an ideal student candidate is a critical issue in the landscape of health sciences education. The issue of selection–that is, predicting who will succeed in medical training and ultimately become a competent professional–is as significant as the curriculum itself. In view of the high-stakes nature of the profession and its impact on the health and well-being of society, ensuring that selection methods are robust and effective is one of the most important tasks of medical education institutions. This not only sets the precedent for the caliber of health professionals prior to entering the medical curriculum, but also touches upon broader societal issues regarding accessibility to a medical career [1–4].

Research on the admission criteria for medical professionals has been conducted from multiple perspectives over the years [1, 3, 4]. Academic ability, represented by metrics such as grade point average (GPA) and medical college admission test (MCAT) scores, has long been a basic criterion for admission in this field [5, 6]. Over time, however, it has been argued that academic ability is a commendable but imperfect predictor of the actual performance upon admission, with one study reporting that academic ability accounts for approximately 23% of the variance in undergraduate medical training outcomes and 6% in postgraduate performance [7]. A growing number of studies have emphasized that students with only high academic ability cannot be transformed into competent medical professionals through medical training; there are other qualities that candidates should possess prior to entering medical training [8].

Since the 1950s and later, the Edinburgh Declaration, the significance of personal character has attracted increasing attention in both academic and practical discourse [1, 9, 10]. Numerous studies have listed characteristics such as integrity and ethics, self-management, strong interpersonal and teamwork skills [11–14]; emotional management and self-motivation skills [15–17]; and empathy [18–21]. International associations and societies of medical education have attempted to delineate the personal competencies expected of medical school entrants. Besides the investigations into personal competencies by the Association of American Medical Colleges (AAMC) in the 1970s and 1990s, admissions and academic affairs officers from U.S. and Canadian institutions rated the importance of specific personal characteristics for success in medical schools and shared insights into their schools’ admission procedures [22]. A recent survey of program directors by the AAMC not only reaffirms the importance of an applicant’s personal attributes over their medical knowledge, but also highlights dissatisfaction with the reliable tools available to measure these competencies [23]. Notably, uniformly applying a set of admission criteria suggested by research or organizations across educational institutions can be challenging, and achieving consensus on these standards within educational entities may be equally difficult [1, 4, 22, 24].

Since the 1960s, with the rising institutional awareness of societal needs, medical schools have begun to consider an admission trend referred to as *holistic review*, which comprises balance among academic readiness, personal and demographic characteristics, experiences, institutional missions and goals, and the institutional stance towards societal needs [24, 25]. The holistic review is different from previous approaches in terms of its emphasis on the link between the review criteria and *institutional goals* [1, 26–30]. This alignment begins with an institutional mission and message for prospective applicants and the broader community [31]. The authority and responsibility of student admissions and subsequent education of the accepted students lie with educational institutions, making it imperative to establish admission criteria that align with the institutions’ mission. The process involves considering and assigning balanced weight to various applicant factors, including academic ability, personal character, attributes aligned with institutional values, and other aspects pointed out by relevant studies and medical education associations. As a result of this process, a distinct set of admission criteria for the ideal candidate can be established, effectively articulated in clear competency descriptions.

Developing distinctive competency criteria for prospective candidates offers several advantages to educational institutions. First, the institution-specific competency criteria for student candidates provide clear guidelines that serve as a vital foundation for an evidence-based selection process. By delineating the competencies of ideal candidates, institutions can ensure that their criteria align with their mission and vision. This alignment facilitates a mission-based, value-aware, evidence-driven, and strategically adaptable admissions process. By ensuring that the admission criteria are well defined, the process not only becomes transparent and fair but also promotes equitable access to medical education for healthcare careers. Second, admission competency criteria can be used to develop a curriculum for graduation competency. These include the qualities of medical professionals that the curriculum should and need not cover for them to attain graduation competency. Some of these qualities may be better cultivated during education, whether through curricular or extracurricular interventions. By defining entrance competencies, educational institutions can distinguish between qualities that students should already possess upon entry and those that institutions aim to instill or enhance. This distinction provides crucial direction in setting realistic educational objectives.

Despite the recognized advantages of institution-specific entrance competencies in health sciences education practices and research, few schools have established *entrance competency* criteria, although the majority possess their own *graduation competency* models [8, 22, 32]. The selection of students in health sciences schools, encompassing fields such as medicine, dentistry, nursing, and other allied health professions, is a complex issue. Some research even describes the selection process as “as much art as science.” (p. 1472) [1]. Creating institution-specific entrance competency standards remains a daunting challenge for educators. Moreover, there is a significant lack of research on this process [4, 32]. Thus, this study develops and validates institution-specific admission competency criteria, based on a case of a dental school in East Asia, and proposes an actionable process for delineating clear descriptions of applicants in alignment with institutional goals and mission that can be generalized for use in other health sciences schools. Additionally, by comparing the resulting criteria with international competency models, we provide a potential reference for future institutional studies and contribute theoretical and practical perspectives to the admission tasks of the health sciences education community.

## Method

To develop and validate the admission competency model, both qualitative and quantitative analyses were conducted in two phases, as summarized in Table 1. Phase I involved constructing an admission competency model using a qualitative method, and phase II involved validating the model using a quantitative method. The purpose of this methodology is to support qualitative findings from selected samples using quantitative results from a broader sample [33]. The research procedure was approved by the institutional review boards of the participating school (#S-D20220035).

**Table 1:**
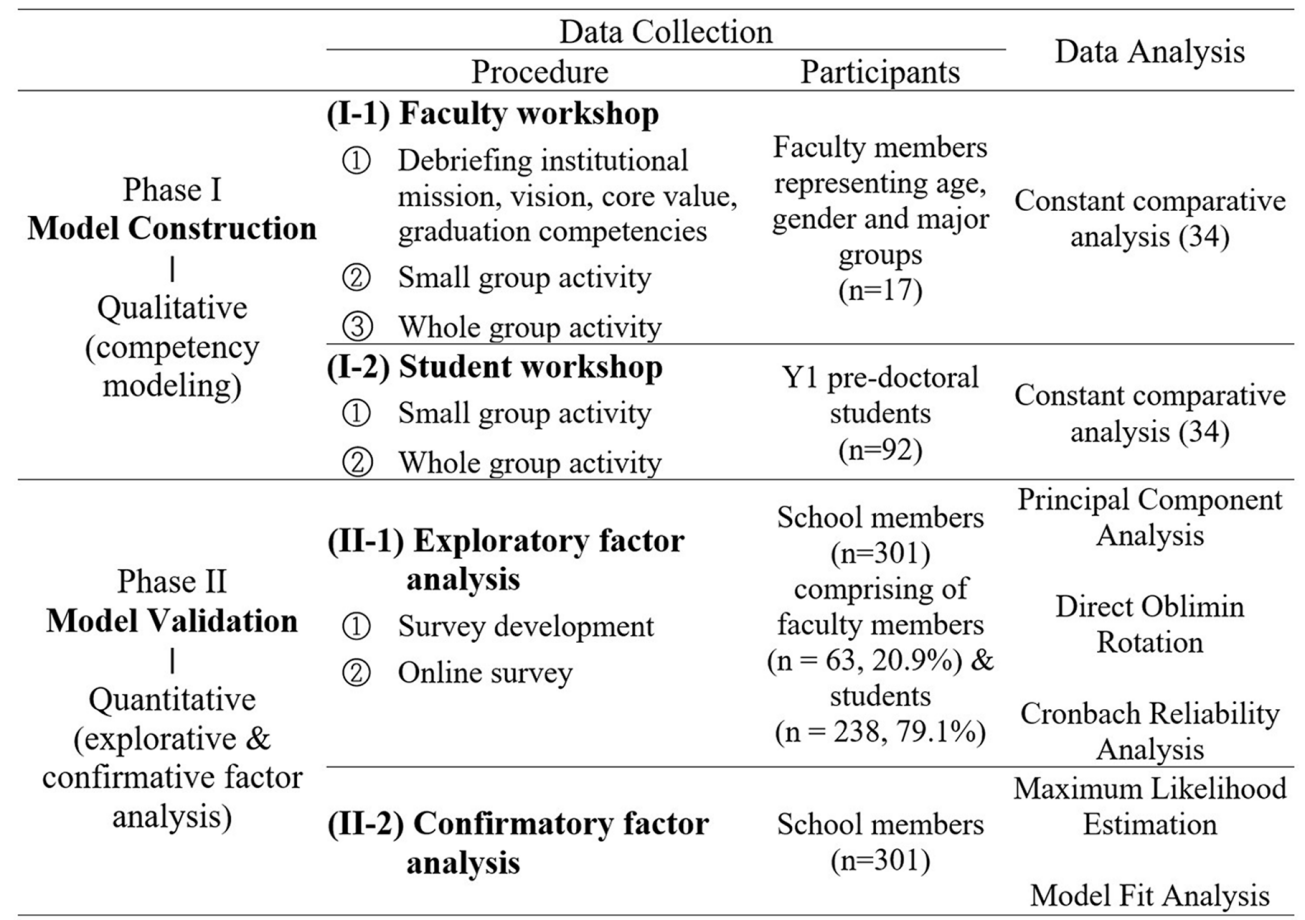
Research Procedure

### Phase 1: model construction

#### Data collection

*Context.* The study was conducted at a top-tier dental school in South Korea, which offers a Doctor of Dental Surgery (DDS) program with approximately 500 students and 100 faculty members. The program trains general dental practitioners (GPs) through a dual academic system with 3 + 4 and 4 + 4 tracks, providing a competency-based curriculum similar to other health sciences programs. The current admissions process includes a document screening to assess applicants’ academic ability and a multiple mini-interview (MMI) to evaluate their personal qualities related to general ethical values.

##### Faculty workshop

*Participants.* Seventeen faculty members in a dental school located in East Asia participated in the workshop, selected to represent variation in age, gender, and discipline: 30s (5.9%, *n* = 1), 40s (52.9%, *n* = 9); 50s (35.3%, *n* = 6), and 60s (5.9%, *n* = 1); female (41.2%, *n* = 7); basic biomedical dentistry (35.3%, *n* = 6), clinical dentistry (41.2%, *n* = 7), and humanities and social dentistry (23.5%, *n* = 4).

*Procedure.* A face-to-face 2-hour workshop titled *Developing Admission Competency* was held. In order to derive competencies aligned with the mission of the institution, needs of stakeholders, and contemporary educational trends, the workshop facilitator initially presented the mission and goals of the institution, as well as the graduation competencies. The workshop also included participants who were well-acquainted with these aspects, specifically the Vice Dean of Academic Affairs and professors from the Department of Dental Education. The workshop involved small group activities and whole group discussions. In the small-group session, four groups of 4 to 5 faculty members were formed. In each group, participants were asked to individually brainstorm the attributes that prospective dental students should have, write the ideas on post-it notes, and share and discuss them with other members of the group. In the subsequent whole group session, the attributes generated by each group were posted on the walls of the room. The participants jointly classified the attributes into several categories. The outcomes of each small group activity and the whole group discussions were visually and verbally recorded.

##### Student workshop

*Participants*. Ninety-two first year pre-doctoral dental students participated in the workshop, and 15 groups of six to seven students were formed. The pre-doctoral stage in dental education is the level of education that prepares students to become general dental practitioners upon graduation, similar to Undergraduate Medical Education (UME) for medical students. Among the participants, 44.2% were female (*n* = 42), and the majority were in their 20s (97.8%, *n* = 90) and two in their 30s (2.2%).

*Procedure*. Online student activities in both small and large groups were implemented during the *Introduction to Clinical Dentistry 1* course. This course aims to develop the clinical and behavioral competencies required of future dental professionals while familiarizing them with the clinical environment. Online tools such as Zoom, Google Jamboard, and Google Sheets were used to encourage students to actively participate in online discussions. In the small-group session, students joined the group meetings on Zoom and used Google Jamboard to exchange their opinions on the attributes that they should possess, after which they classified the attributes into several categories. During the whole group session, each group presented their outcomes on Zoom. Prior to their presentation, each group was asked to write down the attributes for prospective dental students based on categories on a Google Sheets document so that ideas of all the groups could be collected in one file.

#### Data analyses

The attributes and competencies for prospective dental students identified during the workshops were refined and extracted using a constant comparative analysis. Constant comparative analysis is a method frequently used for analyzing qualitative data, where data are compared and categories emerge or are integrated together [34]. First, we refined the attributes for prospective dental students from outcomes of the faculty and student workshops. All attributes were transformed into noun forms, and those with the same meaning were unified into one term. Thereafter, attributes with similar meanings were grouped together and competencies were named for each group. Data saturation was achieved at this point, as further data collection and analysis yielded no new attributes or competencies, suggesting a sufficiently comprehensive data set to ensure robust findings. In naming the competencies, we referenced the premed competencies for entering medical students of AAMC [35] the attributes of medical professionalism noted by Cruess and Cruess [36], and competencies for the new general dental education under the American Dental Education Association (ADEA) [37]. The attributes within and between the categories were thereafter compared to move them to other categories. These modifications were repeated throughout the entire analysis. Finally, we described the behavioral properties of each attribute. The authors worked both independently and jointly, and disagreements were discussed and resolved by consensus.

### Phase 2: model validation

#### Data collection

We developed a questionnaire using items from the previous two workshops. In the beginning of phase 2, we defined the admission competency model as:

*a set of attributes*, *skills or competencies of an entering dental student in order to be successful in the pre-doctoral training and later in their dental professional career*,*which may be better equipped prior to start of the curriculum rather than be heavily fostered through the curriculum*.

We determined 10 constructs and listed 47 potential items for each construct. The questionnaire asked respondents to rate the degree of importance of 47 attributes for prospective dental students on a 5-point Likert scale (1 = least important to 5 = very important) that was administered on Google Forms for a month (See Appendix 1). A total of 301 individuals participated in the survey, comprising 20.9% faculty members (*n* = 63) and 79.1% students (*n* = 238) with 60.5% male (*n* = 182) and 39.5% female (*n* = 119). This sample represents 58.9% of all faculty (*N* = 107) and 48.6% of all students (*N* = 490). 43.5% of the participants were under 26 years old (*n* = 131), 36.5% were aged 26 to 35 (*n* = 110), 5.6% were aged 36 to 45 (*n* = 17), 8.3% were aged 46 to 55 (*n* = 25), and 6.0% were over 55 years old (*n* = 18). The age distribution within the faculty subgroup, with 4.8% under 36, 27.0% aged 36 to 45, 39.7% aged 46 to 55, and 28.6% over 55, reflects the overall faculty composition, ensuring a representative sample. Of them, 49.2% were basic biomedical dentistry majors (*n* = 31) and 50.8% clinical dentistry majors (*n* = 32). Among the students, 21.4% were pre-dental (*n* = 51), 42.0% were in Y1 and Y2 (*n* = 100), and 36.6% were in Y3 and Y4 (*n* = 87), ensuring balanced participation across all grade levels.

#### Data analyses

##### Exploratory factor analysis

To identify the factor structure of the admissions competency model, an Exploratory Factor Analysis (EFA), principal component analysis, was conducted using IBM SPSS Statistics with Version 28 (Armonk, New York, USA) on the 47 items with Direct Oblimin Rotation, assuming correlations among factors. The Kaiser–Meyer–Olkin (KMO) measure (KMO = 0.91) and Bartlett’s test of sphericity (χ^2^ (1081) = 7749.95, *p* < 0.001) confirmed the adequacy of the sample for factor analysis. Preliminary data analyses and screening were performed with a set of criteria: factor loading higher than 0.4 and Cronbach’s alpha higher than 0.6.

##### Confirmatory factor analysis

To examine construct validity, 301 responses were subjected to Confirmatory Factor Analysis (CFA) using IBM SPSS Statistics AMOS Version 28 (Armonk, NY, USA) to verify whether the data fit well with the proposed factors of the EFA. The path coefficients were estimated using maximum likelihood estimation and the model fit was assessed using a set of fit indices.

## Results

### Phase I results: attributes and competencies for prospective dental students

Forty-seven attributes were listed and described with their operational definitions, which were then thematically classified into ten competency categories, as shown in Table 2. The ten competencies for prospective dental students were *personal/moral integrity*, *persistence*, *innovative mindset*, *professionalism*, *social skills*, *integrated self-management skills*, *leadership and insight*, *self-directed/lifelong learning*, *analytical and creative thinking*, and *physical and practical skills.* Of these, six competencies—analytical and creative thinking, social skills, integrated self-management skills, self-directed/lifelong learning, physical and practical skills, and leadership and insight—pertain to the domain of skills, and four competencies—innovative mindset, personal/moral integrity, professionalism, and persistence—pertain to the domain of attitudes and values. The knowledge domain was not included in analysis.

**Table 2:**
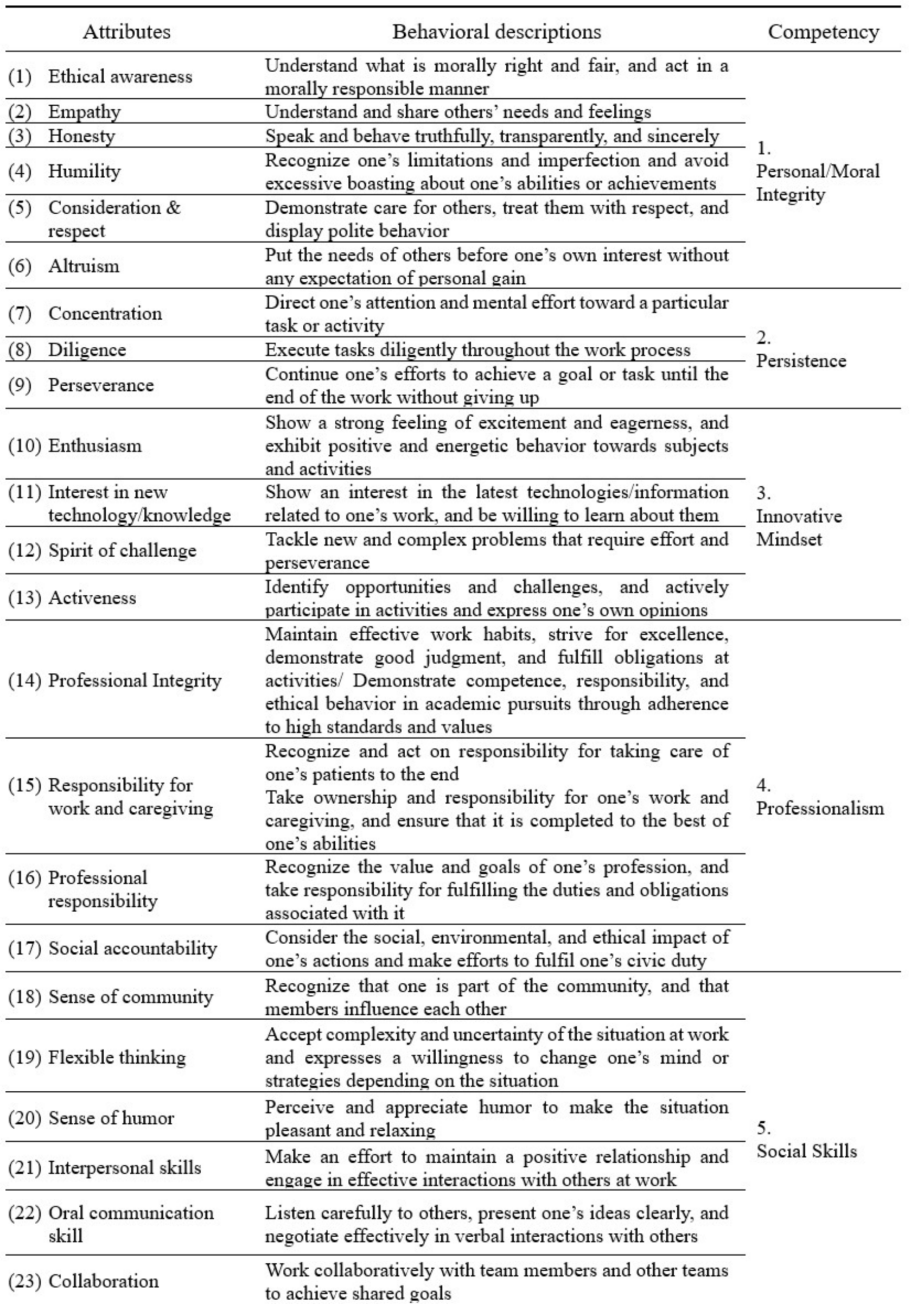

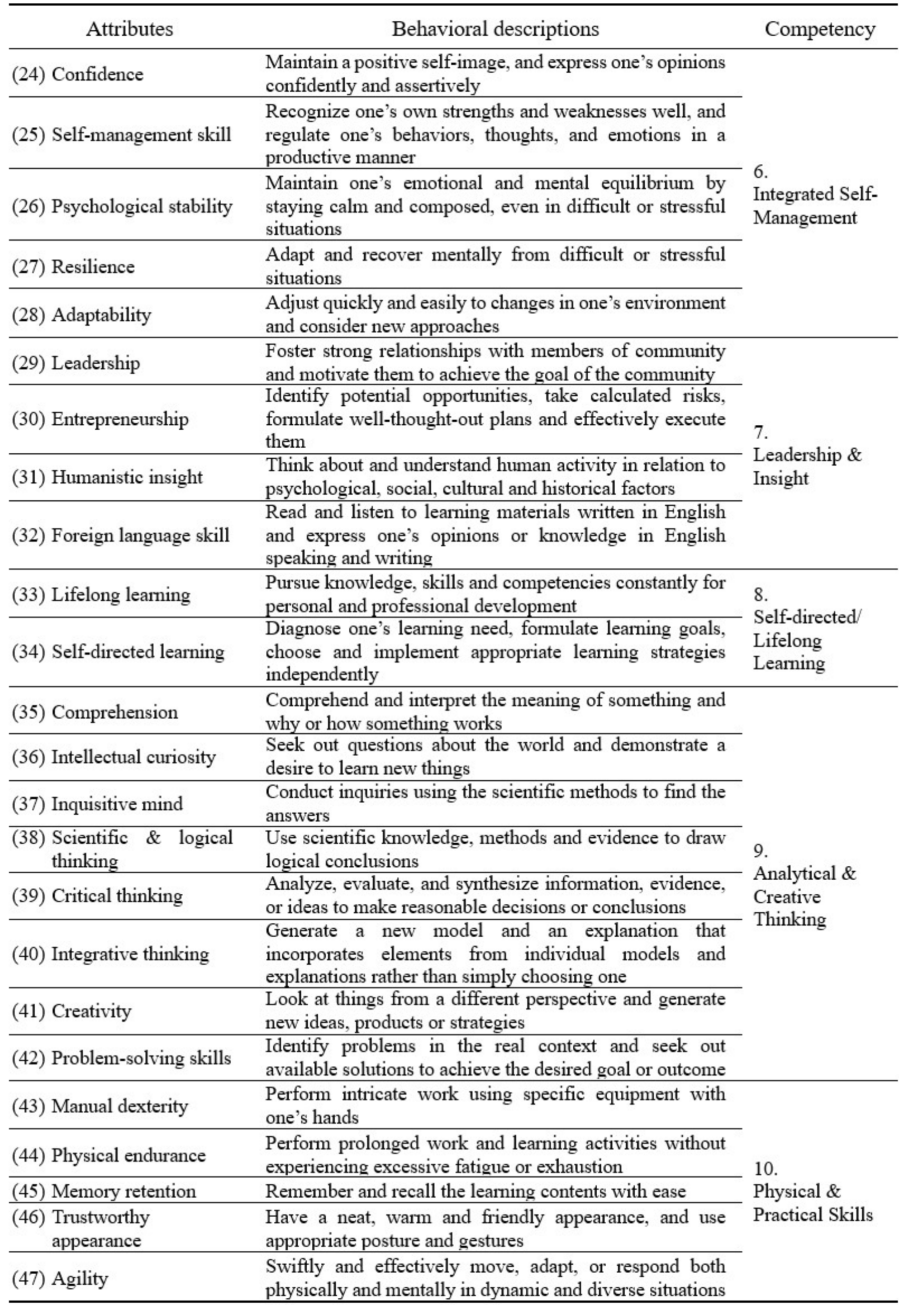
Attributes/competencies for prospective dental students

### Phase II results: validated admission competencies

#### Exploratory factor analysis (EFA)

EFA was conducted on 47 attributes, yielding 10 factors. Two criteria were applied to determine the factors: first, factor loadings below 0.40 in the structure matrix were suppressed; second, if attributes had factor loadings with a slight difference across multiple factors, they were assigned to the factor with more plausible characteristics. Rotated factor loadings ranged from │0.411│ to │0.858│; four items with absolute factor loading in their │0.4│ range—entrepreneurship (0.493), problem-solving skills (-0.471), psychological stability (0.470), and sense of humor (-0.411)—were retained after serious deliberation. The reliability of the constructs, a result of Cronbach’s alpha analyses ranged from 0.68 to 0.88, with overall α of 0.95. The variances explained by each factor ranged from 2.20 to 31.18%, and the ten-factor solution was highly interpretable, accounting for a cumulative variance of 64.13%, as shown in Table 3.

**Table 3:** Exploratory factor analysis results: Factor loadings of 47 attributes

Attributes	Factor									
	1	2	3	4	5	6	7	8	9	10
Enthusiasm	0.822									
Activeness	0.802									
Spirit of challenge	0.704									
Interest in new technology and knowledge	0.551									
Scientific & logical thinking		− 0.858								
Critical thinking		− 0.826								
Intellectual curiosity		− 0.792								
Inquisitive mind		− 0.780								
Integrative thinking		− 0.748								
Comprehension		− 0.748								
Creativity		− 0.692								
Problem-solving skills		− 0.471								
Ethical awareness			0.729							
Consideration & respect			0.727							
Altruism			0.723							
Empathy			0.695							
Honesty			0.667							
Humility			0.600							
Professional integrity				0.782						
Responsibility work/care				0.694						
Professional responsibility				0.678						
Social accountability				0.502						
Social skills					− 0.804					
Collaboration					− 0.784					
Oral communication skill					− 0.686					
Sense of community					− 0.535					
Flexible thinking					− 0.513					
Sense of humor					− 0.411					
Diligence						0.806				
Perseverance						0.584				
Concentration						0.463				
Adaptability							0.772			
Confidence							0.762			
Resilience							0.689			
Self-management skill							0.617			
Psychological stability							0.470			
Lifelong learning								− 0.787		
Self-directed learning								− 0.741		
Manual dexterity									0.761	
Physical endurance									0.746	
Memory retention									0.711	
Trustworthy appearance									0.687	
Agility									0.553	
Leadership										0.582
Foreign language ability										0.573
Humanistic insight										0.537
Entrepreneurship										0.493
Eigenvalue	14.65	3.37	2.71	1.80	1.56	1.40	1.30	1.23	1.09	1.03
Variance explained	31.18	7.17	5.77	3.83	3.32	2.98	2.76	2.61	2.31	2.20
Variance explained cum	31.18	38.35	44.12	47.95	51.27	54.26	57.01	59.63	61.94	64.13
Cronbach’s α	0.83	0.88	0.78	0.73	0.75	0.68	0.79	0.73	0.78	0.75

#### Confirmatory factor analysis (CFA)

CFA was conducted using a cross-validation sample (*n* = 301) of 47 attributes organized into 10 factors (constructs). The CFA results revealed that the ten-factor model of the competency criteria provided an acceptable level of fit to the data, suggesting that the model was appropriate for assessing the competency criteria. The path diagram shows significant estimates in every path, and indices indicate a good model [*χ*^*2*^*/df* = 1.369; Bollen-Stein *p* < 0.05; CFI = 0.956, NFI = 0.858, IFI = 0.995, TLI = 0.944, RMSEA = 0.035] (Table 4).

**Table 4:** CFA results with fit indices

Fit Index	Cutoff Criteria	This model
χ^2^/df	< 3.0 (good fit), < 5.0 (acceptable fit),	1.369
Bollen-Stein *p*	> 0.05 (acceptable fit)	0.493
RMSEA	< 0.03 (excellent fit), < 0 0.06 (good fit),	0.035
NFI	> 0.95 (very good fit), > 0.90 (acceptable fit)	0.858
CFI	> 0.95 (very good fit), > 0.90 (acceptable fit)	0.956
TLI	> 0.95 (very good fit), > 0 0.90 (acceptable fit)	0.944

RMSEA: a root mean square error of approximation, NFI: normed fit index, CFI: comparative fit index, TLI: Tucker-Lewis index

## Discussion

The identification and articulation of admission competencies are critical for both the selection process of prospective students and structuring of the curriculum within health sciences schools. Through a well-defined framework of admission competencies, it is feasible to develop suitable admission-evaluation methodologies encompassing standardized performance assessments, multiple mini-interviews, and various tests, such as written examinations, essays, and evaluations of manual dexterity. Furthermore, the establishment of admission competencies facilitates the precise definition of the requisite knowledge and skills for successful graduation [38–41].

The construction of admission competencies is inherently dynamic, continuously evolving, and adaptable to meet the shifting societal expectations of the health professions. Conventionally, admission competencies have been the purview of authoritative entities such as health sciences education associations and institutional admission committees [22]. However, this study advocates a more inclusive approach that actively involves academic stakeholders in developing admission competencies. This collaborative strategy can foster a comprehensive and nuanced understanding of the diverse skills and qualities required in the contemporary field of health professions, including dentistry in this study. It promotes direct and participatory engagement among those deeply involved in the educational process, thereby fostering a sense of ownership among students and faculty members [41, 42]. This methodological shift seeks to equip entering students with a better understanding of the dynamic and evolving demands of their future professional roles.

We identified ten competencies and 47 attributes of prospective dental students. Table 5 provides a summary comparing these competencies with the 17 Premed Competencies for Entering Medical Students of AAMC [35]. The AAMC Premed competencies are generally aligned with those derived from this study; however, distinct attributes specific to this educational institution were identified. Competencies such as *Social Skills*, *Personal/Moral Integrity*, *Professionalism*, *Self-directed/Lifelong Learning*, and *Analytical and Creative Thinking* were common to both organizations. Among these, *professionalism* or *service orientation* is commonly emphasized for healthcare providers who care for patients [4, 37, 43, 44]. *Patient care* involves multiple intricate steps, often requiring time for the therapeutic effects to be achieved. Students must care for their patients with integrity and professional responsibility [45]. However, teaching these values through 4–5 years of dental education programs alone has become an exceedingly challenging endeavor because adult learners have individualized value systems from diverse experiential backgrounds [46]. Therefore, educational experts and admission committees should develop effective measures to evaluate the pre-professional features of students at admission. Considering the potential societal harm that can arise from individuals entering a profession without professional values, the significance of these competencies becomes even more pronounced.

**Table 5:**
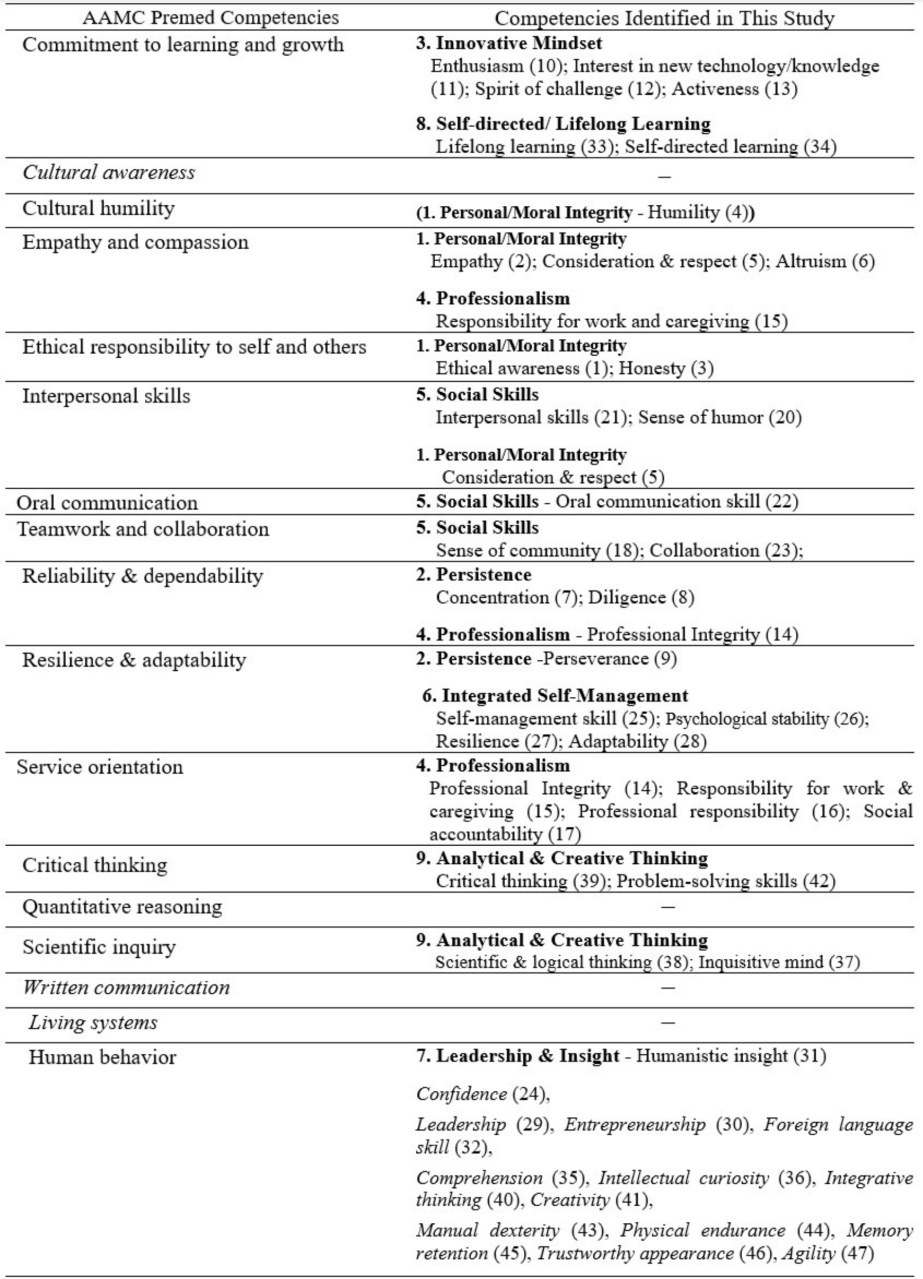
Comparison between resulting competencies and AAMC Premed competencies

Notably, present in AAMC competencies but absent from this institution were *Cultural awareness*, *Cultural humility*, *Quantitative reasoning*, *Written communication*, and *Living systems.* First, *Cultural awareness* and *Cultural humility* did not emerge as relevant competencies in this institution. However, this institution includes general humility as one of its attributes, possibly because of its affiliation with a homogeneous cultural context with limited racial diversity. Cultural competence extends beyond race and includes the ability to understand individuals with various social and cultural differences, recognize biases, and collaborate effectively with others. As societal conflicts, including intergenerational disputes and gender-related issues, have intensified, there is an increasing demand for healthcare professionals to adeptly navigate and address these conflicts to establish positive relationships with patients [47]. Although cultural competency may not be an immediate concern for the institution in this study, it is worth considering the proactive inclusion of Diversity, Equity, and Inclusion (DEI) awareness and competency in the near future.

Second, one of the unmentioned competencies, *written communication* is also significant for healthcare professionals [48, 49]. Some medical schools include educational programs on patient note writing from the pre-clerkship period [50, 51]. Writing patient notes is evaluated in terms of documentation, differential diagnosis, justification, workup, and composite. Medical students’ scores on patient note-writing tasks during the Objective Structured Clinical Examination (OSCE) were significantly related to clinical performance [52, 53]. As writing patient notes is the first step in clinical reasoning to make an appropriate differential diagnosis, the demand for written communication training among students entering health sciences school is reasonable and has been emphasized [52]. Moreover, written communication is fundamental to interpersonal relationships in patient care. Kripalani et al. [54] reported that delayed or inaccurate communication between physicians negatively affects patient care and contributes to adverse events.

AAMC’s Commitment to *Learning and Growth* resembles Self-directed/Lifelong Learning; however, this institution appears to seek candidates with a more challenging mindset, reflected in the third competency of *Innovative Mindset* with attributes of *Enthusiasm; Interest in new technology/knowledge; Spirit of challenge* and *Activeness*. This competency reflects the competitive dental practice environment, where modern dentists demand the adoption of cutting-edge technologies in their practice, making openness to new technologies a key competency [55].

*Physical and Practical Skills* is one of the competencies specific to dentistry. Kao et al. applied a wire-bending exercise to test manual dexterity, which was positively correlated with laboratory examination test scores [54]. The Canadian dental admission test (DAT) involves perceptual reasoning as well as manual dexterity tests (MDT), such as the chalk carving test [56]. Although chalk carving scores for testing hand skills significantly correlate with preclinical operative dentistry performance, the MDT score has not been reported to be an accurate predictor of final class standing [57, 58]. Rather, Cleghorn et al. [59] suggested a cutoff value for MDT, as MDT scores of 10 or less showed extremely weak psychomotor skills.^58^ Although MDT has not been applied for admission selection, faculty and students perceived manual dexterity as a helpful skill for performance in dental schools and future careers. Though *Scientific & logical thinking* and *Critical thinking* correspond to *Quantitative reasoning*, they were not directly addressed. Regarding *Living systems*, this institution did not require prerequisite knowledge; instead, it included *Memory retention* as a practical competency.

Unique to this institution are *Confidence*, *Leadership*, *Entrepreneurship*, *Foreign language skill*, *Comprehension*, *Intellectual curiosity*, *Integrative thinking*, *Creativity*, *Manual dexterity*, *Physical endurance*, *Memory retention*, *Trustworthy appearance*, and *Agility*. While valuing innovative minds, leadership, and creativity, it is clear that both the practical demands of the field of dentistry and the goal of the institution to achieve global eminence in this field are reflected. This comparison suggests that rather than using standardized competencies, an institution could develop a tailored set of competencies reflecting its unique institutional and field-specific needs, highlighting the importance of building institution-specific competencies following the methodology proposed in this study.

Table 6 offers a comparative analysis of admission and graduation competencies at the institution studied, revealing both significant differences and overlaps. Unique admission competencies such as A1 (personal/moral integrity), A2 (persistence), A3 (innovative mindset), A6 (integrated self-management), A7 (leadership & insight), A8 (self-directed/lifelong learning), and A10 (physical & practical skills) lay a critical foundation for incoming students. Some attributes (A2, A6, A8, A10) are essential for the rigorous demands of their training, while others (A1, A3, A7) are often inherent traits that are challenging to cultivate solely through education, leading the institution to favor applicants who naturally possess these traits. Admission competencies A4 (professionalism), A5 (social skills), and A9 (analytical & creative thinking) correspond to graduation competencies G1 (professionalism as a health provider), G2 (communication skills), and G3 (knowledge-based critical thinking). These competencies, foundational at entry, are continuously deepened and specialized throughout the educational journey, culminating in more sophisticated, profession-specific attributes as students grows. The graduation competencies of G4 (examination, diagnosis, treatment planning), G5 (clinical competency), and G6 (practice management and administration) build upon the foundational attributes to foster holistic dental professionals. This progression exemplifies the curriculum’s structured development from broad admission competencies to detailed, specialized graduation competencies, equipping students with essential foundational skills and advanced professional capabilities. Clarifying which competencies are expected before admission, which are continuously deepened or specialized from admission through graduation, and which are newly developed during the educational program can lead to a curriculum that is more effectively designed for their ultimate professional success.

**Table 6:**
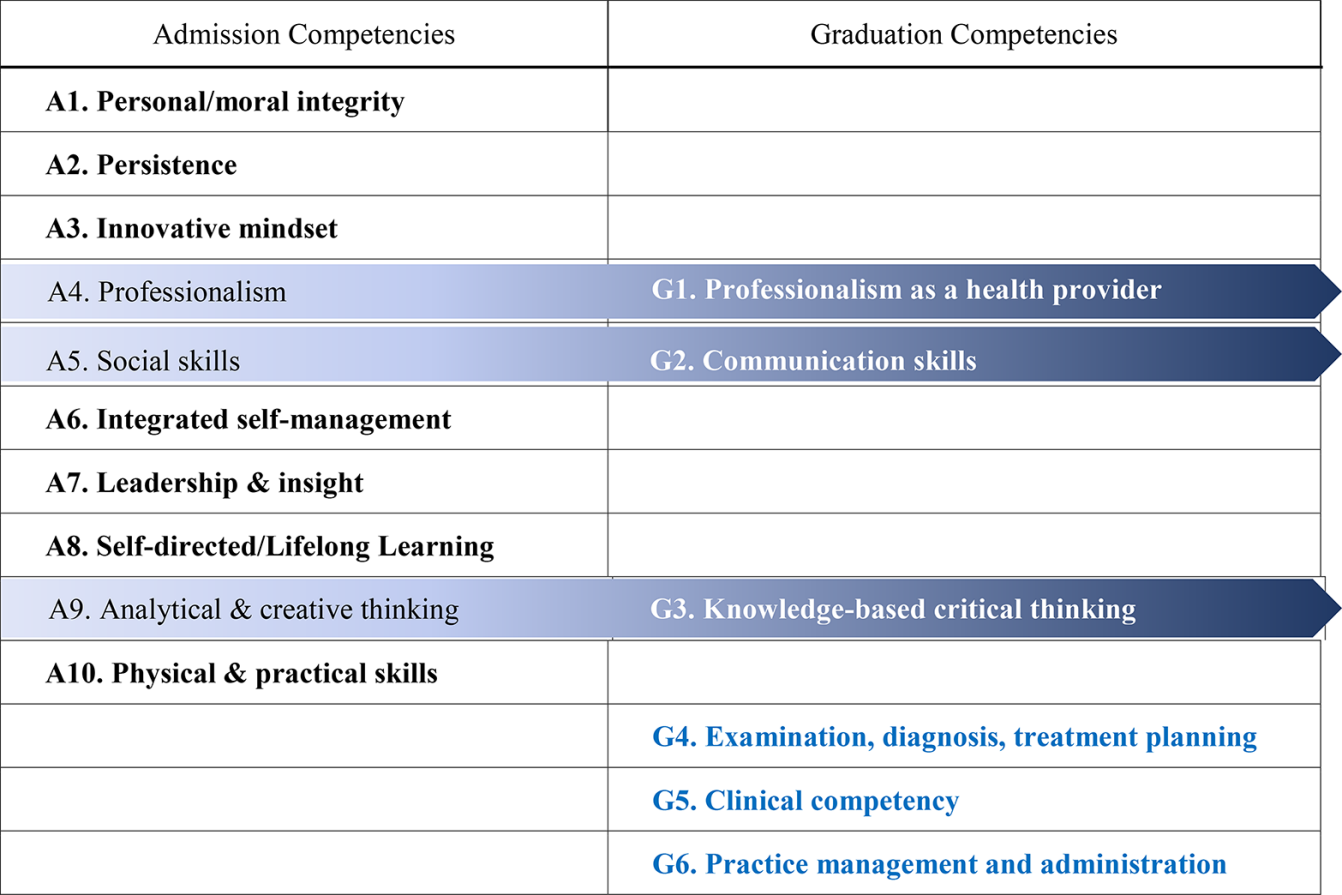
Comparison between admission and graduation competencies of the institution

This study presents a compilation of the attributes and competencies of dental students admitted by an educational institution. However, since the study reports on a single dental education institution in South Korea, the generalization of the findings may be limited, depending on how different other institutions are from the context provided. Nonetheless, the described process could potentially be useful for other health sciences institutions.

Despite our comprehensive efforts to ensure accurate classification of attributes through expert consensus and rigorous statistical analysis, we acknowledge the limitation that some attributes may overlap across multiple competencies. This overlap reflects the inherent complexity and interconnectedness of the competencies and attributes. Future research should aim to further refine their naming and definitions and clarify their conceptual distinctions.

This study developed admission competencies based on insights from students and faculty. To better reflect societal demands of the profession, future research should incorporate perspectives from recent graduates and real-world practice experiences. However, achieving consensus on the ideal qualities of prospective students among members of an institution is practically very challenging. Future research exploring differing opinions among students, faculty, and alumni can help develop a more rational and democratic model for selecting candidates, promoting better communication and shared understanding.

The study does not specify the relative importance of each attribute or competency. Future research should address specific attributes and competencies that could be emphasized or excluded, considering various institutional factors. Most importantly, as with all educational interventions, it is crucial to conduct long-term, longitudinal studies to evaluate the effectiveness of the new admission practice proposed in this study by comparing it with the previous selection method. Such research will undoubtedly contribute significantly to improving the scientific and systematic approaches to evidence-based student selection.

## Conclusions

This study underscores the importance of developing institution-specific admission competencies that reflect each institution’s mission and vision, showcasing the methodology employed. By involving both faculty and students in the development process, it identified a set of competencies distinct from the AAMC-Premed competencies, catering to the dynamic and evolving demands of the profession within its social context. Developed admission competencies must be intricately linked to graduation competencies, underscoring the necessity for the comprehensive integration of these competencies into the curriculum. This approach is vital for ensuring that students are not only admitted based on their potential but also guided towards the successful completion of their education and entry into the profession. It is hoped that this study will aid health science education institutions in developing tailored admission competencies, paving the way for a more comprehensive and effective model of student selection that is mission-based, value-aware, evidence-driven, and strategically adaptable.

## Data Availability

Related data of this study can be available upon request to the corresponding author.

## Appendix 1

Survey on Admission Competencies for Prospective Dental Students. The following attributes are the possible qualities we hope students entering our school should possess. Please indicate how important you think each attribute is using the following scale

**Table:**

Attributes	Least important ←—→ Very important				
Empathy	1	2	3	4	5
Consideration & respect	1	2	3	4	5
Humility	1	2	3	4	5
Ethical awareness	1	2	3	4	5
Altruism	1	2	3	4	5
Honesty	1	2	3	4	5
Diligence	1	2	3	4	5
Responsibility for work and caregiving	1	2	3	4	5
Professional responsibility	1	2	3	4	5
Professional Integrity	1	2	3	4	5
Social accountability	1	2	3	4	5
Psychological stability	1	2	3	4	5
Resilience	1	2	3	4	5
Confidence	1	2	3	4	5
Adaptability	1	2	3	4	5
Self-management skill	1	2	3	4	5
Sense of community	1	2	3	4	5
Leadership	1	2	3	4	5
Collaboration	1	2	3	4	5
Interpersonal skills	1	2	3	4	5
Oral communication skill	1	2	3	4	5
Sense of humor	1	2	3	4	5
Memory retention	1	2	3	4	5
Comprehension	1	2	3	4	5
Scientific & logical thinking	1	2	3	4	5
Critical thinking	1	2	3	4	5
Integrative thinking	1	2	3	4	5
Agility	1	2	3	4	5
Problem-solving skills	1	2	3	4	5
Intellectual curiosity	1	2	3	4	5
Inquisitive mind	1	2	3	4	5
Perseverance	1	2	3	4	5
Concentration	1	2	3	4	5
Humanistic insight	1	2	3	4	5
Flexible thinking	1	2	3	4	5
Manual dexterity	1	2	3	4	5
Physical endurance	1	2	3	4	5
Creativity	1	2	3	4	5
Activeness	1	2	3	4	5
Enthusiasm	1	2	3	4	5
Interest in new technology/knowledge	1	2	3	4	5
Spirit of challenge	1	2	3	4	5
Lifelong learning	1	2	3	4	5
Self-directed learning	1	2	3	4	5
Entrepreneurship	1	2	3	4	5
Foreign language skill	1	2	3	4	5
Trustworthy appearance	1	2	3	4	5

## Abbreviations

GPA: Grade Point Average
MCAT: Medical College Admission Test
AAMC: Association of American Medical Colleges
ADEA: American Dental Education Association
EFA: Exploratory Factor Analysis
CFA: Confirmatory Factor Analysis
KMO: Kaiser–Meyer–Olkin
DEI: Diversity, Equity, and Inclusion
OSCE: Objective Structured Clinical Examination
DAT: Canadian Dental Admission Test
MDT: Manual Dexterity Test
